# The Prognostic Value of Brain Dysfunction in Critically Ill Patients with and without Sepsis: A *Post Hoc* Analysis of the ICON Audit

**DOI:** 10.3390/brainsci11050530

**Published:** 2021-04-23

**Authors:** Ilaria A. Crippa, Fabio S. Taccone, Xavier Wittebole, Ignacio Martin-Loeches, Mary E. Schroeder, Bruno François, Katarzyna Kotfis, Silvio A. Ñamendys-Silva, Xavier Forceville, Jordi Solé-Violán, Luis E. Fontes, Jean-Louis Vincent, on behalf of the ICON Investigators

**Affiliations:** 1 Department of Intensive Care, Erasme Hospital, Université Libre de Bruxelles, 1070 Brussels, Belgium; ilaria.alice.crippa@gmail.com (I.A.C.); ftaccone@ulb.ac.be (F.S.T.); 2 Department of Critical Care, Cliniques Universitaires St Luc, UCLouvain, 1200 Brussels, Belgium; xavier.wittebole@uclouvain.be; 3 Department of Clinical Medicine, Trinity Centre for Health Sciences, Multidisciplinary Intensive Care Research Organization (MICRO), Wellcome Trust, HRB Clinical Research, St James’s University Hospital Dublin, D08 NHY1 Dublin, Ireland; drmartinloeches@gmail.com; 4 Hospital Clinic, IDIBAPS, Universidad de Barcelona, Ciberes, 08036 Barcelona, Spain; 5 Division of Trauma and Critical Care Surgery, Medical College of Wisconsin/Froedtert Hospital, Milwaukee, WI 53226, USA; libby823@gmail.com; 6 Intensive Care Unit and Inserm CIC 1435 & UMR 1092, Dupuytren University Hospital, 87000 Limoges, France; realim@unilim.fr; 7 Department of Anesthesiology, Intensive Therapy and Acute Intoxications, Pomeranian Medical University, 70-111 Szczecin, Poland; katarzyna.kotfis@pum.edu.pl; 8 Department of Critical Care Medicine, Instituto Nacional de Cancerología, Ciudad de México 14080, Mexico; snamendys@incan.edu.mx; 9 Division of Pulmonary, Anesthesia and Critical Care Medicine, Instituto Nacional de Ciencias Médicas y Nutrición Salvador Zubirán, Ciudad de México 14080, Mexico; 10 Medico-Surgical Intensive Care Unit, Great Hospital of East Francilien—Meaux Site, Hôpital Saint Faron, 77100 Meaux, France; xforceville@invivo.edu; 11 Clinical Investigation Center (CIC Inserm 1414), CHU de Rennes—Université de Rennes, 35033 Rennes, France; 12 Intensive Care Unit, University Hospital of GC Dr Negrín, 35010 Las Palmas, Gran Canaria, Spain; jsolvio@gobiernodecanarias.org; 13 Departamento de Medicina Baseada em Evidências, Medicina Intensiva, Urgência e Emergência—Faculdade de Medicina de Petrópolis, 25680-120 Petrópolis, Brazil; luis.fontes@ceo2.com.br

**Keywords:** brain function, Glasgow Coma Scale, ICU, outcome

## Abstract

Brain dysfunction is associated with poor outcome in critically ill patients. In a post hoc analysis of the Intensive Care over Nations (ICON) database, we investigated the effect of brain dysfunction on hospital mortality in critically ill patients. Brain failure was defined as a neurological sequential organ failure assessment (nSOFA) score of 3–4, based on the assumed Glasgow Coma Scale (GCS) score. Multivariable analyses were performed to assess the independent roles of nSOFA and change in nSOFA from admission to day 3 (ΔnSOFA) for predicting hospital mortality. Data from 7192 (2096 septic and 5096 non-septic) patients were analyzed. Septic patients were more likely than non-septic patients to have brain failure on admission (434/2095 (21%) vs. 617/4665 (13%), *p* < 0.001) and during the ICU stay (625/2063 (30%) vs. 736/4665 (16%), *p* < 0.001). The presence of sepsis (RR 1.66 (1.31–2.09)), brain failure (RR 4.85 (3.33–7.07)), and both together (RR 5.61 (3.93–8.00)) were associated with an increased risk of in-hospital death, but nSOFA was not. In the 3280 (46%) patients in whom ΔnSOFA was available, sepsis (RR 2.42 (1.62–3.60)), brain function deterioration (RR 6.97 (3.71–13.08)), and the two together (RR 10.24 (5.93–17.67)) were associated with an increased risk of in-hospital death, whereas improvement in brain function was not.

## 1. Introduction

Acute brain dysfunction remains one of the most frequent neurological complications in critically ill patients [1] and is associated with high mortality rates, poor quality of life, and long-term neurological sequelae among survivors [2,3]. Sepsis-associated encephalopathy (SAE) is a diffuse cerebral dysfunction that accompanies sepsis in the absence of direct central nervous system involvement or other possible causes (e.g., structural central nervous or systemic metabolic conditions) [4]. The broad term, SAE, can cover a wide range of different types of brain dysfunction, including delirium, coma, and non-convulsive seizures, and it is one of the most common sepsis-related organ dysfunctions and often the first to manifest [5,6]. The pathophysiology of SAE is still unclear, and it involves several mechanisms, including diffuse neuroinflammation, excitotoxicity, microglial activation, alterations in neurotransmission, and cerebral ischemia, which can be aggravated by extra-cerebral factors, such as hypoxemia or stress hyperglycemia [7].

Several studies have reported a worse prognosis in patients with SAE compared to other septic patients. In a study of 232 intensive care unit (ICU) patients, those with SAE had significantly higher 28-day mortality rates than those without [8]. In another study of 50 septic patients, mortality was correlated to the severity of SAE graded by the Glasgow Coma Scale (GCS) score [9]. In a large prospective multicenter database of 2513 patients with sepsis on ICU admission, even mild alteration of mental status (i.e., GCS 13–14) was an independent predictor of ICU mortality after adjustment for several confounders [10]. Nevertheless, it remains unclear whether brain dysfunction per se is a determinant of poor outcome or whether the combined presence of sepsis enhances the negative effects of brain dysfunction on patient survival. Moreover, as SAE can develop at any stage of sepsis and its duration can be quite variable, assessment of changes in brain function over time might be more important than a single evaluation (e.g., the worst GCS during the ICU stay). In particular, comatose patients showing subsequent neurological recovery may have a different prognosis than those with a persistently low or decreasing GCS.

Therefore, the hypothesis of this study was that clinically diagnosed brain dysfunction combined with sepsis would have a greater impact on in-hospital mortality than sepsis or brain dysfunction alone. We also hypothesized that a lack of improvement in neurological dysfunction over the first few days of the ICU stay would be associated with worse in-hospital mortality rates compared to improved neurological function.

## 2. Materials and Methods

### 2.1. Study Design

This study is a post hoc analysis of the prospective, multicenter, observational Intensive Care over Nations (ICON) audit, which was designed to assess the epidemiology and outcome of critically ill patients worldwide [11]. Briefly, all patients older than 16 years admitted to a participating ICU (listed in the Supplementary Appendix) during the two-week period between 8 May and 18 May 2012 were included in the study. Institutional ethical review board approval was obtained by each participating institution in accordance with local regulations. Patients admitted to the ICU for less than 24 h for routine postoperative surveillance, and patients readmitted during the study period were excluded. Data were anonymously collected using electronic case report forms via a secured internet-based website. For the purposes of this analysis, patients with a primary brain injury (e.g., head trauma, stroke, post-anoxic coma, central nervous system infection) as the reason for ICU admission were excluded.

### 2.2. Data Management

Data collected on admission included demographics, reason for admission, and comorbidities. Clinical and laboratory data for calculation of the Simplified Acute Physiology (SAPS) II and Acute Physiology And Chronic Health Evaluation (APACHE) II scores were reported as the worst values within the first 24 h after admission. A daily evaluation of organ function was performed using the sequential organ failure assessment (SOFA) score [12]; for the neurological component of SOFA (nSOFA, based on the GCS value), participants were requested to report either the “actual” GCS or the “assumed” GCS, i.e., the value the patient would have without sedation. However, whether the reported GCS was the actual or the assumed one was not reported in the database. The presence of microbiological and clinical infections, as well as antibiotic treatment, was recorded daily. Data were collected daily for up to 28 days of ICU stay. Outcome data were collected at the time of ICU and hospital discharge or at 60 days.

### 2.3. Definitions

Details of all the definitions used in the ICON audit have been published previously [11]. Infection was defined according to the criteria of the International Sepsis Forum. Sepsis was defined as the presence of infection with the concomitant occurrence of at least one organ failure. Organ failure was defined as a SOFA sub-score >2 for the organ in question [12]. Septic shock was defined as sepsis associated with a cardiovascular SOFA >2. Neurological condition on admission and during the ICU stay (the worst value) was assessed using the nSOFA score; brain dysfunction was defined as an nSOFA >0 (i.e., GCS ≤ 14); and brain failure was defined as an nSOFA of 3–4 (i.e., GCS ≤ 9). Changes in nSOFA over time (ΔnSOFA) were calculated as the difference between nSOFA on day 3 and nSOFA on admission. For the purposes of this analysis, patients were categorized as having deteriorated, unchanged, or improved brain function according to the ΔnSOFA value (Table 1).

### 2.4. Statistical Analysis

The original audit data were processed in the Department of Intensive Care of Erasme Hospital, Brussels. Descriptive statistics were computed for all study variables. No imputation for missing values was used. The Kolmogorov–Smirnov test was used, and histograms and normal-quantile plots were examined to verify the normality of distribution of continuous variables. Discrete variables are expressed as counts (percentage) and continuous variables as means ± standard deviation (SD) or median [25th–75th percentiles]. Differences between groups (hospital survivors and non-survivors) were assessed using the analysis of variance, Kruskal–Wallis test, Student’s *t* test, Mann–Whitney test, χ^2^ test, or Fisher’s exact test as appropriate; variables with *p* < 0.2 were included in the multivariable analysis. Collinearity between variables was checked by inspection of the correlation between them, by looking at the correlation matrix of the estimated parameters, and by looking at the change of parameter estimates and at their estimated standard errors (SEs). Q-Q plots were drawn to check for normality in the residuals. Sepsis, nSOFA (on admission and the worst during the ICU stay), and brain failure were included in the multivariable model and adjusted risk ratios (RRs) with their 95% confidence intervals (CIs) were computed, using patients without sepsis and with no brain failure as reference. A second multivariable analysis was conducted only in patients with available ΔnSOFA values; variables with *p* < 0.2 in the univariate analysis were included in the multivariable model. Collinearity and normality were assessed as previously described. Sepsis and unchanged, improved, or deteriorated brain function were included in the multivariable model, and adjusted risk ratios (RRs) with their 95% CIs were computed, using patients without sepsis and with no brain dysfunction as reference. Data were analyzed using IBM SPSS Statistics software, version 24 for Windows and R software, version 2.0.1 (CRAN project). All reported *p* values are 2-sided, and *p* < 0.05 was considered to indicate statistical significance.

## 3. Results

Data from 7192 (2096 septic and 5096 non-septic) patients without primary brain injury were included in the analysis. Among the septic patients, 830 (40%) had septic shock on admission and 159 (8%) developed septic shock during their ICU stay. Characteristics of the study population are reported in Table 2.

### 3.1. Neurological Function and Outcome

**Figure 1:**
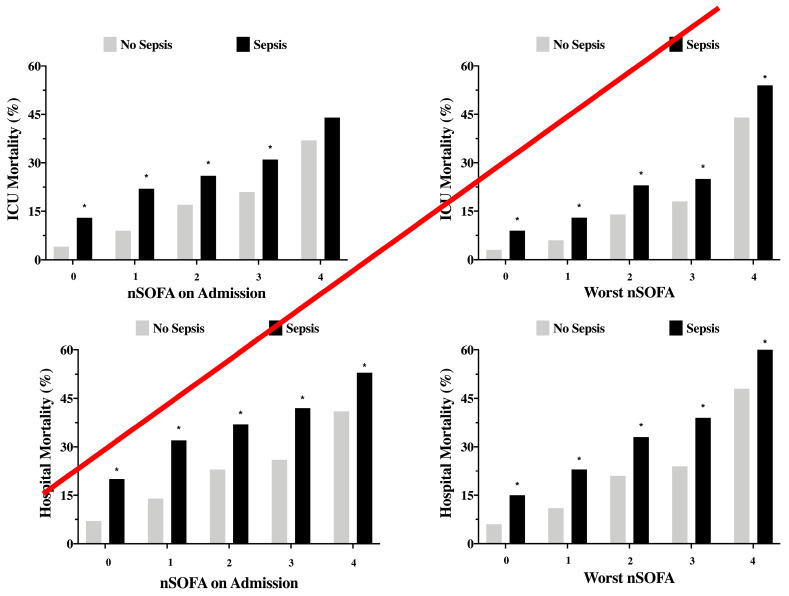
Intensive care unit (ICU) and hospital mortality in septic and non-septic patients, according to the neurological sequential organ failure assessment (nSOFA) score on admission and the worst nSOFA during the ICU stay. * *p* < 0.05.

nSOFA values on admission and during the ICU stay were available for 2095 (100%) septic and 4665 (92%) non-septic patients. The admission nSOFA and the worst nSOFA during the ICU stay were higher in septic than in non-septic patients (0 (0–2) vs. 0 (0–1) and 1 (0–3) vs. 0 (0–1), respectively, both *p* < 0.001 (Supplemental Figures S1 and S2)). Septic patients were more likely than non-septic patients to have brain failure on admission (434/2095 (21%) vs. 617/4665 (13%), *p* < 0.001) and during the ICU stay (625/2063 (30%) vs. 736/4665 (16%), *p* < 0.001). ICU and hospital mortality rates increased with increasing admission and worst nSOFA scores during the ICU stay in septic and non-septic patients and were greater in septic than in non-septic patients, except for patients with an nSOFA = 4 on admission in whom ICU mortality was similar in septic and non-septic patients (Table 2 and Figure 1).

**Figure 2:**
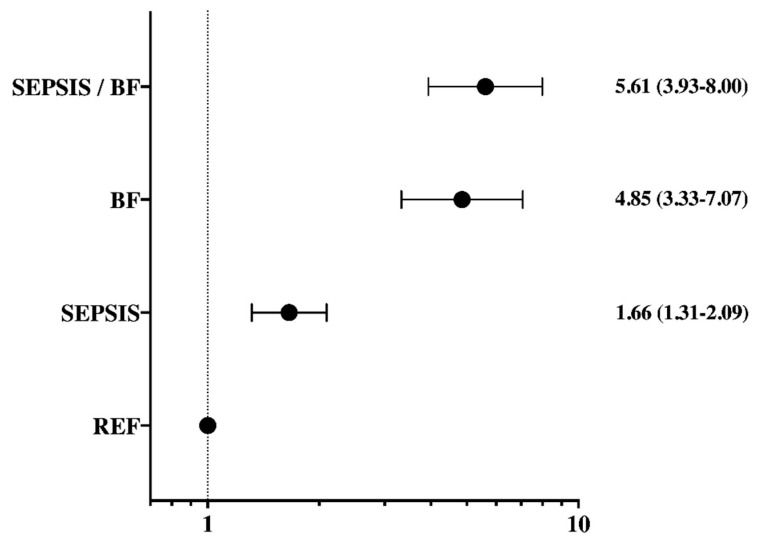
Adjusted risk of hospital mortality (multivariable analysis) according to the presence of sepsis and/or brain failure (BF), using non-septic patients without brain failure as reference (REF). Data are expressed as adjusted risk ratios and 95% confidence intervals.

Hospital mortality was greater in septic patients with brain failure (318/604 (53%)) than in other patients (non-septic with brain failure 267/702 (38%); sepsis, no brain failure 275/1397 (20%); no sepsis, no brain failure 334/3878 (9%); *p* < 0.001—Supplemental Figure S3). In the multivariable analysis including type of hospital, age, sex, SAPS II and SOFA scores, type of admission, comorbidities (chronic obstructive pulmonary disease, diabetes mellitus, cancer, liver cirrhosis, immunosuppression, chronic heart, and renal failure), non-neurological organ failure, and the use of mechanical ventilation and renal replacement therapy, sepsis (adjusted RR 1.66 (1.31–2.09)), brain failure (adjusted RR 4.85 (3.33–7.07)), and the combination of sepsis and brain failure (adjusted RR 5.61 (3.93–8.00)) were independently associated with an increased risk of in-hospital death (Figure 2); nSOFA was not independently associated with hospital mortality.

### 3.2. Evolution of Neurological Function and Outcome

ΔnSOFA was available for 3280 (46%) patients: 1758 (54%) patients had no brain dysfunction, 582 (18%) had unchanged brain function, 705 (21%) had improved brain function, and 235 (7%) had brain function deterioration. Brain function deterioration was more common in septic than in non-septic patients (134/1401 (10%) vs. 101/1879 (5%), *p* < 0.001). By contrast, there were no significant differences in the proportion of patients with and without sepsis with improved brain function (318/1401 (23%) vs. 387/1879 (21%), *p* = 0.15). Absolute in-hospital mortality in all these subgroups is shown in Supplemental Figure S4.

**Figure 3:**
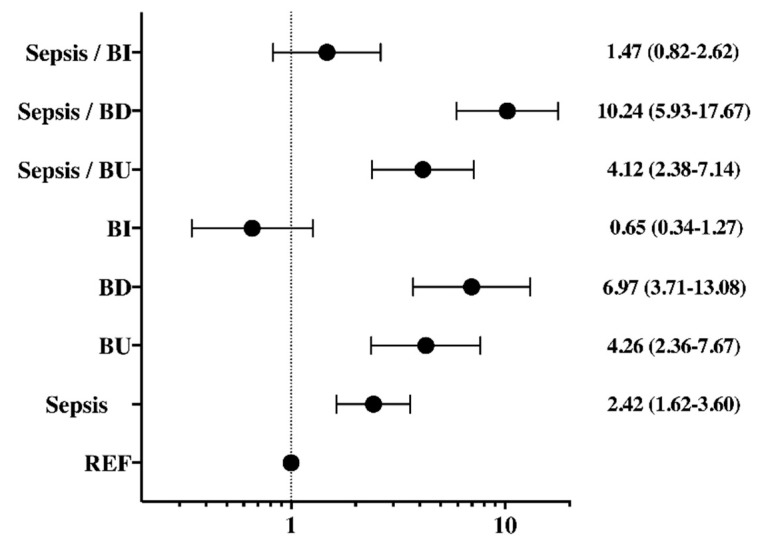
Adjusted risk of hospital mortality (multivariable analysis) according to the presence of sepsis and/or unchanged brain function (BU), brain function improvement (BI), or brain function deterioration (BD), using non-septic patients without brain dysfunction as reference (REF). Data are expressed as adjusted risk ratios and 95% confidence intervals.

In the multivariable analysis, sepsis (adjusted RR 2.42 (1.62–3.60)) and brain function deterioration were independently associated with an increased risk of in-hospital death (adjusted RR 6.97 (3.71–13.08)), and this risk was greater when sepsis was also present (adjusted RR 10.24 (5.93–17.67)) (Figure 3). Improved brain function was not independently associated with a reduced risk of in-hospital mortality (adjusted RR 0.65 (0.34–1.27)), even when sepsis was also present (adjusted RR 1.47 (0.82–2.62)).

## 4. Discussion

Our results, obtained from a large international cohort of septic and non-septic critically ill patients, can be summarized as follows: (1) brain dysfunction, assessed by the GCS, was common in ICU patients and more frequent in patients with sepsis than in those without; (2) worse neurological status was associated with greater hospital mortality; (3) brain dysfunction was independently associated with an increased risk of in-hospital death, and the risk was higher in patients with sepsis than in those without.

Our results are consistent with previous studies. In a small study of 50 septic patients, Eidelman et al. showed that ICU mortality progressively increased with the severity of brain dysfunction evaluated by GCS [9]. In a retrospective study of 2533 septic patients, the presence of shock together with hypoxemia and altered mental status was associated with the highest mortality in septic patients, even after adjustment for the use of mechanical ventilation [13]. Zhang et al. showed that septic patients with clinically diagnosed SAE had higher mortality than those without brain dysfunction [8]. Finally, in a large cohort of septic and non-septic patients (*n* = 1333), an acutely altered mental status due to sepsis carried a relative risk of mortality higher than that of normal mental status, and it was even higher than that associated with pre-existing brain dysfunction [3].

However, previously published studies included the neurological assessment on admission [13,14] or the worst neurological assessment at a pre-defined period of time after admission [8,9,15]. In our study, we found that only brain failure (i.e., GCS 3–9), and not mild alterations in brain function, was independently associated with hospital mortality. This finding may be explained by the large number of variables included in the multivariable model, which may have blunted the potential effects of small changes in GCS on patient outcomes. Moreover, GCS is a measure of global brain dysfunction and other clinical scales, such as the Confusion Assessment Method for the ICU (CAM-ICU) or Intensive Care Delirium Screening Checklist (ICDSC), may be more accurate to assess mild alteration or transient changes in brain function in critically ill patients [16,17]. Interestingly, the association of sepsis and brain failure further increased the risk of death, suggesting that severe brain dysfunction has an independent prognostic role in septic patients. However, GCS is not effective at detecting the presence of delirium, which remains a powerful predictor of outcome in ICU patients, in particular among those treated with mechanical ventilation [18]. Large prospective studies using predefined diagnostic approaches for delirium and SAE diagnosis could help to further clarify the prognostic value of these conditions in patients with sepsis.

As neurological status in the critically ill patient typically fluctuates over time and SAE is a potentially reversible condition, single time-point evaluations may not provide an accurate reflection of the actual neurological condition of critically ill patients. As such, changes in neurological function could provide additional information. In a study of 352 critically ill patients, repeated measurement of SOFA score over time correlated with outcome in critically ill patients: regardless of the initial SOFA score, the mortality was 50% when the SOFA score increased, 27% to 35% when the score was unchanged, and less than 27% when the SOFA score decreased during the first 96 h after admission [19]. In a cohort of 2619 critically ill patients, a 1-point increase in total SOFA score between day 1 and day 3 after admission was associated with an increased risk of mortality [14]. We showed that deterioration in neurological condition during the first 3 days after ICU admission was associated with increased mortality in both septic and non-septic patients. Notably, improved brain function was associated with the same risk of death as that in non-septic patients without brain dysfunction. This is a novel and important finding, which should be included in future studies evaluating the role of SAE on the outcome of ICU patients.

Our study has several strengths. First, it included a large, international cohort of patients, increasing the generalizability of our findings. Second, it is the first to assess the effects of brain dysfunction on hospital mortality in septic and non-septic patients. Third, a large number of predictive variables was available to adjust the risk of death in the multivariable model. However, there are also some limitations. Similar to previous studies [10], we were unable to identify the reasons for brain dysfunction in this study; the pathophysiology of SAE is complex and multifactorial [7]. Some patients may have experienced secondary brain injury (e.g., acute ischemic stroke, cerebral hemorrhage, seizures), which might have further aggravated their brain function, independent of the initial reason for ICU admission [10]. Second, patients with sepsis often have concomitant metabolic conditions that could have influenced their neurological status [20]. Third, half of the study population did not have an nSOFA on day 3 (either missing or available only later during the ICU stay), which may have significantly biased our observations; in particular, early ICU discharge or death may have removed the less severe and the sickest patients of the entire cohort. As a result of even more missing values for other days (e.g., day 5 or 7), we could not assess whether changes in neurological function using another time-point would have provided the same information. Fourth, staff members were supposed to record the assumed GCS, independent of the use of sedation, but this was not checked [21,22]. Fifth, the design of the ICON audit assumed a single daily assessment of brain function using the GCS score, which may not be sensitive enough to identify transient mild or moderate brain dysfunction. Future cohort studies should include a repeated (at least every 12 h) evaluation of brain dysfunction and should also include a dedicated delirium screening tool.

## 5. Conclusions

This post hoc analysis on a large, international cohort of critically ill patients showed that hospital mortality was significantly higher in patients with brain failure than in those without, particularly in the presence of sepsis. Early changes in brain function also provided prognostic information, particularly in patients with sepsis, suggesting the need for repeated neurological assessment in future studies on SAE.

## Figures and Tables

**Table 1:** Definitions of brain function according to changes in neurological sequential organ failure assessment (ΔnSOFA) score from admission to day 3.

Admission nSOFA	Day 3 nSOFA	ΔnSOFA	Brain Condition
0	0	0	unchanged
0	1/2/3/4	1/2/3/4	deterioration
1	0	−1	improvement
1	1	0	unchanged
1	2/3/4	1/2/3	deterioration
2	0/1	−2/−1	improvement
2	2	0	unchanged
2	3/4	1/2	deterioration
3	0/1	−3/−2	improvement
3	2/3/4	−1/0/1	unchanged
4	0/1/2	−4/−3/−2	improvement
4	3/4	−1/0	unchanged

**Table 2:** Demographics, organ failure, and mortality rate in patients with and without sepsis.

	Sepsis	No Sepsis	*p* Value
Age (years)	61 (±17) (*n* = 2089)	60 (±18) (*n* = 5054)	<0.001
SAPS II score	46 (±17)	35 (±17)	<0.001
SOFA score	7 (±4)	5 (±4)	<0.001
ICU LOS (days)	4 [2–9] (*n* = 2036)	2 [1–4] (*n* = 4803)	<0.001
Hospital LOS (days)	12 [6–25] (*n* = 2001)	8 [4–14] (*n* = 4557)	<0.001
Male, n/total (%)	2989/5020 (60)	1227/2079 (59)	0.7
Type of admission	(*n* = 2006)	(*n* = 4769)	
● Medical	1290 (64)	2605 (55)	<0.001
● Surgical	663 (33)	1902 (40)	<0.001
● Trauma	45 (2)	229 (5)	<0.001
Admission source			
● Operating room	308 (15)	1085 (21)	<0.001
● Emergency room	689 (33)	1851 (36)	0.006
● Other hospital	200 (10)	431 (9)	0.14
● Same hospital	745 (36)	1211 (24)	<0.001
COPD	355 (17)	578 (11)	<0.001
Cancer	264 (13)	533 (11)	0.009
● Metastatic	97 (5)	157 (3)	0.002
● Hematological	81 (4)	77 (2)	<0.001
Diabetes mellitus	257 (12)	502 (10)	0.003
Liver cirrhosis	105 (5)	170 (3)	0.001
Immunodepression	145 (7)	130 (3)	<0.001
Artificial airway			
● On admission	1092 (52)	1729 (34)	<0.001
● During ICU stay	1293 (62)	1886 (37)	<0.001
Mechanical ventilation			
● On admission	1192 (57)	1823 (36)	<0.001
● During ICU stay	1382 (66)	2000 (39)	<0.001
Renal replacement therapy			
● On admission	342 (11)	261 (5)	<0.001
● During ICU stay	570 (27)	518 (10)	<0.001
Organ failure on admission			
● Respiratory	670 (32)	706 (14)	<0.001
● Coagulation	179 (9)	309 (6)	<0.001
● Hepatic	299 (14)	438 (9)	<0.001
● Neurological	434 (21)	617 (12)	<0.001
● Renal	517 (25)	959 (19)	<0.001
● Circulatory	830 (40)	941 (19)	<0.001
Organ failure during ICU stay			
● Respiratory	961 (46)	947 (19)	<0.001
● Coagulation	358 (17)	454 (9)	<0.001
● Hepatic	641 (31)	841 (17)	<0.001
● Neurological	625 (30)	736 (14)	<0.001
● Renal	1143 (55)	2144 (42)	<0.001
● Circulatory	1064 (51)	1163 (23)	<0.001
ICU mortality according to admission nSOFA *			
● nSOFA = 0	139/1075 (13)	125/3063 (4)	<0.001
● nSOFA = 1	72/323 (22)	49/546 (9)	<0.001
● nSOFA = 2	63/240 (26)	56/327 (17)	0.006
● nSOFA = 3	62/200 (31)	59/282 (21)	0.008
● nSOFA = 4	98/225 (44)	118/320 (37)	0.07
ICU mortality according to maximum nSOFA *			
● nSOFA = 0	75/877 (9)	82/2849 (3)	<0.001
● nSOFA = 1	43/326 (13)	37/625 (6)	<0.001
● nSOFA = 2	56/246 (23)	49/345 (14)	0.005
● nSOFA = 3	61/242 (25)	55/304 (18)	0.03
● nSOFA = 4	199/372 (54)	184/415 (44)	0.006
Hospital mortality according to admission nSOFA **			
● nSOFA = 0	207/1038 (20)	212/2917 (7)	<0.001
● nSOFA = 1	101/315 (32)	74/529 (14)	<0.001
● nSOFA = 2	85/232 (37)	71/311 (23)	<0.001
● nSOFA = 3	82/194 (42)	70/275 (26)	<0.001
● nSOFA = 4	117/221 (53)	128/312 (41)	0.004
Hospital mortality according to maximum nSOFA **			
● nSOFA = 0	125/845 (15)	153/2710 (6)	<0.001
● nSOFA = 1	71/315 (23)	66/606 (11)	<0.001
● nSOFA = 2	78/236 (33)	69/326 (21)	0.001
● nSOFA = 3	93/236 (39)	71/297 (24)	<0.001
● nSOFA = 4	225/368 (61)	196/405 (48)	<0.001

Data are presented as mean (±SD), median (IQRs), or count (%). SOFA = Sequential Organ Failure Assessment; SAPS = Simplified Acute Physiology Score; COPD = chronic obstructive pulmonary disease; nSOFA = neurological SOFA; ICU = intensive care unit: LOS: length of stay * ICU outcome was available for 6601 patients (2063 in the sepsis group and 4538 in the non-sepsis group); ** Hospital outcome was available for 6581 patients (2001 in sepsis and 4580 in non-sepsis group).

## Data Availability

The datasets used during the current study are available from the corresponding author on reasonable request.

## Supplementary Materials

The following are available online at https://www.mdpi.com/article/10.3390/brainsci11050530/s1, Figure S1: Distribution of neurological sequential organ failure assessment (nSOFA) scores on admission to the intensive care unit (ICU) in septic and non-septic patients; Figure S2: Distribution of the worst neurological sequential organ failure assessment (nSOFA) score during the intensive care unit (ICU) stay in septic and non-septic patients; Figure S3: Hospital mortality according to the presence of sepsis with or without brain failure (BF; neurological sequential organ failure assessment (nSOFA) score 3–4). Non-septic patients without brain dysfunction were considered as the reference group (REF); Figure S4: Hospital mortality according to the presence of sepsis with or without unchanged brain function (BU), brain function improvement (BI) or deteriorated brain function (BD). Non-septic patients without brain dysfunction were considered as the reference group (REF); Appendix: Alphabetical list of participating centers by region and country.
