# Supplementary material for: The Prognostic Value of Brain Dysfunction in Critically Ill Patients with and without Sepsis: A Post Hoc Analysis of the ICON Audit
Source: Brain Sci. 2021 Apr 23;11(5):530. doi: 10.3390/brainsci11050530 (PMC8146463; doi:10.3390/brainsci11050530)
Supplement: Supplementary file 1 [file brainsci-11-00530-s001.zip › brainsci-1160547-supplementary.pdf]

The prognostic value of brain dysfunction in in critically ill patients with and without sepsis: a post hoc analysis of the ICON audit

Crippa IA et al

Online Supplement

Supplemental Figure S1. Distribution of neurological sequential organ failure assessment (nSOFA) scores on admission in septic and non-septic patients.

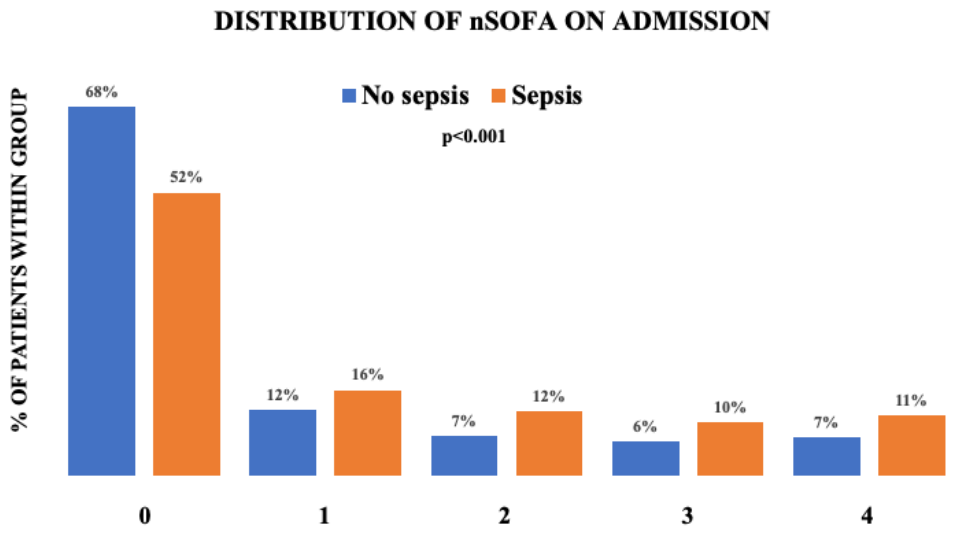

Supplemental Figure S2. Distribution of the worst neurological sequential organ failure assessment (nSOFA) score during the intensive care unit (ICU) stay in septic and non-septic patients

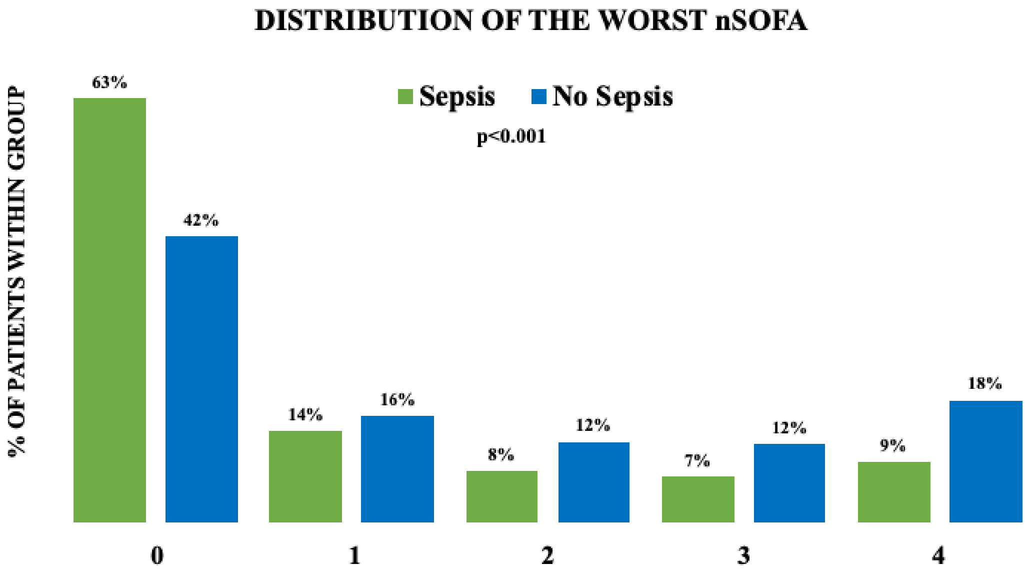

Supplemental Figure S3. Hospital mortality according to the presence of sepsis with or without brain failure (BF; neurological sequential organ failure assessment [nSOFA] score 3-4). Non-septic patients without brain dysfunction were considered as the reference group (REF).

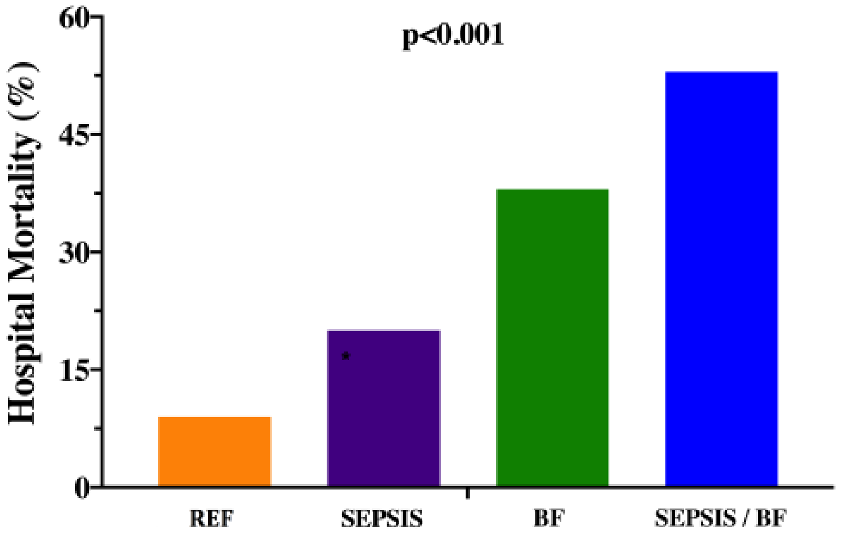

Supplemental Figure S4. Hospital mortality according to the presence of sepsis with or without unchanged brain function (BU), brain function improvement (BI) or deteriorated brain function (BD). Non-septic patients without brain dysfunction were considered as the reference group (REF).

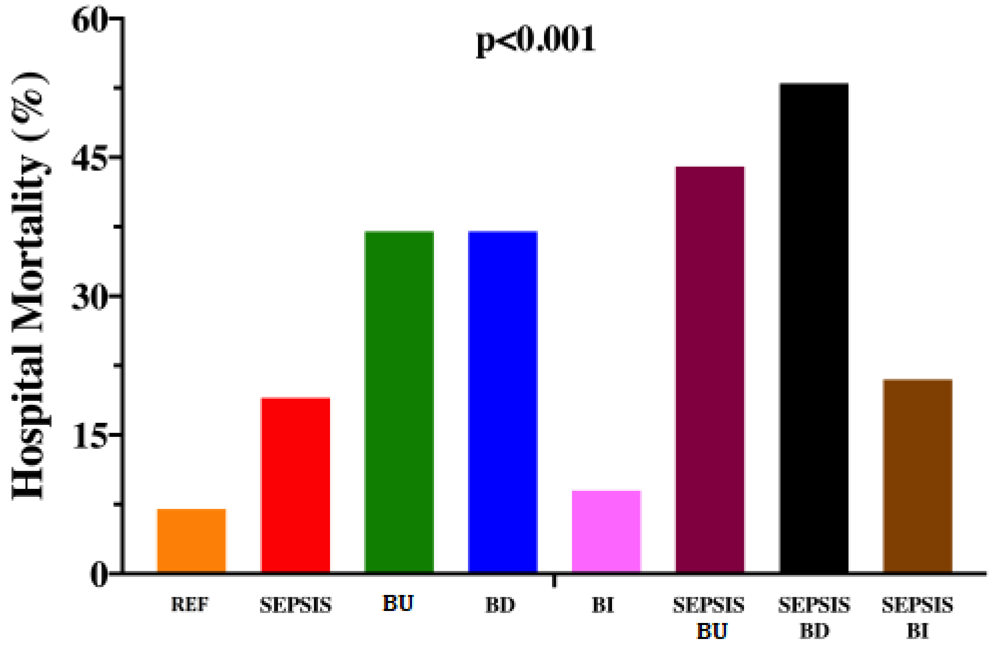

Appendix. Alphabetical list of participating centers by region and country

Africa
Angola: Clinica Sagrada Esperança (E Tomas)
Democratic Republic of Congo: Cliniques Universitaires De Kinshasa (E Amisi Bibonge)
Morocco: Chu Ibn Rochd Casablanca (B Charra); Ibn Sina Hospital (M Faroudy)
South Africa: Chris Hani Baragwanath Academic Hospital (L Doedens); Grey's Hospital (Z Farina); Sandton Medi Clinic (D Adler); Tygerberg Hospital (C Balkema); Union Hospital Alberton (A Kok)
Tunisia: Bizerte Hospital (S Alaya); Military Hospital of Tunis (H Gharsallah)

East Europe
Albania: National Trauma Centre and Military Hospital, Tirana (D Muzha)
Bulgaria: Alexandrovska University Hospital (A Temelkov); Emergency University Hospital 'Pirogov' (G Georgiev); Tokuda Hospital Sofia (G Simeonov); Uh St Ekaterina Sofia (G Tsaryanski); University Hospital for Obstetrics and Gynaecology (S Georgiev); University Hospital Sveta Marina - Varna (A Seliman)
Croatia: General Hosp. Sibenik (S Vrankovic); University Hospital Centre "Sestre Milosrdnice" (Z Vucicevic); University Hospital Centre Zagreb (I Gornik); University Hospital for Infectious Diseases (B Barsic); University Hospital Dubrava (I Husedzinovic)
Czech Republic: Centre of Cardiovascular and Transplant Surgery (P Pavlik); Charles University Hospital (J Manak); IKEM, Prague (E Kieslichova); KNTB Zlín A.S. (R Turek); Krajska Nemocnice Liberec (M Fischer); Masarykova Nemocnice V Usti Nad Labem (R Valkova); St. Anne's University Hospital Brno (L Dadak); University Hospital Haradec Králové (P Dostal); University Hospital Brno (J Malaska); University Hospital Olomouc (R Hajek); University Hospital Plzen (A Židková); Charles University Hospital Plzen (P Lavicka)
Estonia: Tartu University Hospital (J Starkopf)
Georgia: Critical Care Medicine Institute (Z Kheladze); Jo Ann Medical Centre (M Chkhaidze); Kipshidze Central University Hospital (V Kaloiani)
Hungary: Dr. Kenessey Albert Hospital (L Medve); Fejér County St George Teaching Hospital (A Sarkany); Flor Ferenc County Hospital (I Kremer); Jávorszky Ödön Hospital (Z Marjanek); Peterfy Hospital Budapest (P Tamasi)
Latvia: Infectology Centre of Latvia (I Krupnova); Paul Stradins Clinical University Hospital (I Vanags); Riga East Clinical University Hospital (V Liguts)
Lithuania: Hospital of Lithuanian University of Health Sciences Kauno Klinikos (V Pilvinis); Vilnius University Hospital (S Vosylius); Vilnius University Hospital "Santariskiu Clinics", HSICU (G Kekstas); Vilnius University Hospital Santariskiu Clinics, CICU (M Balciunas)
Poland: Csk Mswia (J Kolbusz); Medical University (A Kübler); Medical University Of Wroclaw (B Mielczarek); Medical University Warsaw (M Mikaszewska-Sokolewicz); Pomeranian Medical University (K Kotfis); Regional Hospital in Poznan (B Tamowicz); Szpital Powiatowy W Ostrowi Mazowieckiej (W Sulkowski); University Hospital, Poznam (P Smuszkiewicz); Wojewódzki Szpital Zakazny (A Pihowicz); Wojewódzkie Centrum Medyczne (E Trejnowska)
Romania: Emergency County Hospital Cluj (N Hagau); Emergency Institute for Cardiovascular Diseases (D Filipescu); Fundeni Clinical Institute (G Droc); Galati Hospital (M Lupu); Inbi "Prof. Dr. Matei Bals" (A Nica); Institute of Pulmonology Marius Nasta (R Stoica); Institutul Clinic Fundeni (D Tomescu); Sfantul Pantelimon Hospital (D

Constantinescu); Spitalul Cf 2 Bucuresti (G Valcoreanu Zbaganu); “Luliu Hatieganu” University of Medicine and Pharmacy,
Teaching Hospital of Infectious Diseases, Cluj-Napoca (A Slavcovici)
Russia: City Clinical Hospital No 40 (V Bagin); City Hospital No 40 (D Belsky); Clinical Hospital N.A. N.V.Solovyev (S Palyutin); Emergency Research Institute N.A. Djanelidze (S Shlyapnikov); Federal Research Centre Paediatric Haematology, Oncology and Immunology (D Bikkulova); Prof. V.F. Voino-Yasenetsky Krasnoyarsk State Medical University, Krasnoyarsk Regional Hospital (A Gritsan); Medical Association "Novaya Bolnitsa" (G Natalia); Military Medical Academy (E Makarenko); Novosibirsk Medical University (V Kokhno); Omsk Regional Clinical Hospital (A Tolkach); Railway Hospital of Khabarovsk (E Kokarev); St Alexy Hospital (B Belotserkovskiy); State District Hospital (K Zolotukhin); Vishnevsky Institute of Surgery (V Kulabukhov)
Serbia: Clinic for Cardiac Surgery, Clinical Centre of Serbia (L Soskic); Clinic for Digestive Surgery, Clinical Centre Serbia (I Palibrk); Clinic for Vascular Surgery, Clinical Centre Nis (R Jankovic); Clinical Centre of Serbia (B Jovanovic); Clinical Centre of Serbia (M Pandurovic); Emergency Centre, Clinical Centre of Belgrade (V Bumbasirevic); General University Hospital (B Uljarevic); Military Medical Academy (M Surbatovic); Urology Hospital (N Ladjevic)
Slovakia: District Hospital (G Slobodianiuk); Faculty Hospital (V Sobona); University Hospital Bratislava-Hospital Ruzinov ICU (A Cikova); University Hospital Ruzinov Bratislava (A Gebhardtova)

East & Southeast Asia
China: A Tertiary Hospital (C Jun); Affiliated Hospital of Medical College Qingdao Universty (S Yunbo); Beijing Cancer Hospital ,Beijing Institute for Cancer Research (J Dong); Beijing Chaoyang Hospital (S Feng); Beijing Friendship Hospital (M Duan); Beijing Tongren Hospital Affiliate of Capital Medical University (Y Xu); Beijing University People's Hospital (X Xue); Beijing Luhe Hospital (T Gao); Cancer Hospital, Chinese Academy of Medical Sciences (X Xing); China Academy of Chinese Medical Sciences Guang 'An Men Hospital (X Zhao); Chuxiong, Yunnan Province, People's Hospital (C Li); Donge County People's Hospital of Shandong Province (G Gengxihua); Fu Wai Hospital, Chinese Academy of Medical Sciences (H Tan); Fujian Provincial Hospital (J Xu); Fuxing Hospital, Capital Medicine University (L Jiang); Guangdong General Hospital (Q Tiehe); Henan Provincial People's Hospital (Q Bingyu); Xian Jiaotong University College of Medicine (Q Shi); Kunming Third People's Hospital (Z Lv); Lanzhou University Second Hospital (L Zhang); No 309th Hospital (L Jingtao); No.1 Hospital of China Medical University (Z Zhen); Peking University Shougang Hospital (Z Wang); Peking University Third Hospital (T Wang); Pla Navy General Hospital (L Yuhong); Qilu Hospital Shandong University (Q Zhai); Ruijin Hospital Affiliated Medical School of Jiaotong University, Shanghai (Y Chen); Shandong Provincial Hospital (C Wang); Shanghai 10th People's Hospital (W Jiang); Shanghai First People's Hospital (W Ruilan); Sichuan Provincial People's Hospital (Y Chen); Sichuan Provincial People's Hospital (H Xiaobo); Sir Run Run Shaw Hospital (H Ge); The Affiliated of Guiyang Medical College (T Yan); The Fifth People's Hospital of Shanghai, Fudan University (C Yuhui); The First Affiliated Hospital of Dalian Medical University (J Zhang); The First Affiliated Hospital of Suzhou University (F Jian-Hong); The First Affiliated Hospital of Xinjiang Medical University (H Zhu); The First Hospital of Jilin University (F Huo); The First Hospital of Jilin University (Y Wang); The First People's Hospital of Kunming (C Li); The General Hospital of Shenyang Military Region ,China (M Zhuang); The People’s Hospital of Cangzhou (Z Ma); The Second Hospital of Jilin University (J Sun); The Second People’s Hospital of Liaocheng City Shandong Province (L Liuqingyue); The

Third Xiangya Hospital (M Yang); Tongde Hospital of Zhejiang Province (J Meng); Tongji University Shanghai East Hospital (S Ma); West China Hospital, Scu (Y Kang); Wuhan Centre Hospital (L Yu); Xiangya Hospital, Changsha, Hunan Province, China (Q Peng); Yantai Yuhuangding Hospital (Y Wei); Yantaishan Hospital, Shandong Province (W Zhang); Zhejiang Provincial People's Hospital (R Sun)
Hong Kong (China): Pamela Youde Nethersole Eastern Hospital (A Yeung); Princess Margaret Hospital (W Wan); Queen Elizabeth Hospital (K Sin); United Christian Hospital of Hong Kong SAR (K Lee)
Indonesia: Anestesi (M Wijanti); Pku Muhammadiyah Bantul, Yogyakarta (U Widodo); Rd Mattaher Hospital Jambi (H Samsirun); Rumah Sakit Pantai Indah Kapuk (T Sugiman); Sardjito Hospital (C Wisudarti); School of Medicine Unpad - Hasan Sadikin Hospital (T Maskoen)
Japan: Chiba Hokusoh Hospital, Nippon Medical School (N Hata); Chiba University Hospital (Y Kobe); Fujita Health University School of Medicine (O Nishida); Japanese Red Cross Maebashi Hospital (D Miyazaki); Jichi Medical University Hospital (S Nunomiya); Jikei University School of Medicine (S Uchino); Kimitsu Chuo Hospital (N Kitamura); Kochi Medical School (K Yamashita); Kyoto Prefectural University of Medicine (S Hashimoto); Nara Medical University Hospital (H Fukushima)
Malaysia: Hospital Sultanah Nur Zahirah, Kuala Terengganu, Terengganu, (N Nik Adib); Kuala Lumpur Hospital (L Tai); Queen Elizabeth Hospital 2 (B Tony)
Philippines: Cebu Velez General Hospital (R Bigornia); Chong Hua Hospital (R Bigornia); Perpetual Succour Hospital (R Bigornia); The Medical City (J Palo)
Singapore: Alexandra Hospital (S Chatterjee); National University Health System (B Tan); Singapore General Hospital (A Kong); Tan Tock Seng Hospital (S Goh)
Taiwan: National Taiwan University Hospital (C Lee)
Thailand: Maharaj Nakorn Chiangmai Hospital, Chiaingmai University (C Pothirat); Prince of Songkla University (B Khwannimit); Ramathibodi Hospital (P Theerawit); Ramathibodi Hospital, Somdech Phra Debaratana Medical Centre (P Pornsuriyasak); Siriraj Hospital, Mahidol University (A Piriyapatsom)

Middle East
Egypt: Cairo University (A Mukhtar); Demerdash Surgical Intensive Care Unit (Dsicu); Ain Shams Faculty of Medicine (A Nabil Hamdy); Zaitoun Specialized Hospital (H Hosny)
Iran: Gums (A Ashraf); Imam Hossein Hospital, Sbums (M Mokhtari); Imamreza Hospital (S Nowruzinia); Laleh Hospital (A Lotfi); Shiraz Anesthesiology and Critical Care Research Center (F Zand); Shiraz University of Medical Sciences (R Nikandish); Tehran Medical Sciences University (O Moradi Moghaddam)
Israel: Rabin Medical Centre (J Cohen); Sourasky Tel Aviv Medical Centre (O Sold)
Lebanon: Centre Hospitalier Du Nord (T Sfeir)
Oman: Sohar Hospital (A Hasan)
Palestinian Territories: Specialized Arab Hospital (D Abugaber)
Saudi Arabia: Almana General Hospital (H Ahmad); KFSHRC, Riyadh (T Tantawy); King Abdulaziz Medical City Riyadh (S Baharoom); King Abdulaziz University (H Algethamy); King Saud Medical City (A Amr); Riyadh Military Hospital (G Almekhlafi)
Turkey: Erciyes University Medical Faculty (R Coskun); Erciyes University Medical School (M Sungur); Gülhane Military Medical Academy (A Cosar); International Hospital, Istanbul (B Güçyetmez); Istanbul University Cerrahpasa Medical School Hospital (O Demirkiran); Istanbul University Istanbul Medical Faculty (E Senturk); Karadeniz Technical University, Medical Faculty (H Ulusoy); Memorial Atasehir Hospital (H Atalan); Pamukkale Universty (S Serin); Yuzuncu Yil Universitesy Medical Faculty (I Kati)

United Arab Emirates: Dubai Hospital (Z Alnassrawi); Mafraq Hospital (A Almemari); Sheikh Khalifa Medical City (K Krishnareddy); Tawam Hospital (S Kashef); The City Hospital (A Alsabbah)

North America
Canada: Hôpital Charles Lemoyne (G Poirier); St. Michael's Hospital (J Marshall); Toronto General Hospital (M Herridge); Toronto Western Hospital (M Herridge)
Puerto Rico: San Juan Hospital (R Fernandez-Medero)
United States: Christiana Care Health System (G Fulda); Cincinnati Children's Hospital Medical Centre (S Banschbach); El Camino Hospital (J Quintero); George Washington Hospital (E Schroeder); Hospital of The University of Pennsylvania (C Sicoutris); John H Stroger Hospital of Cook County (R Gueret); Mayo Clinic, CCM (R Kashyap); Mayo Clinic, PCC (P Bauer); Medical College of Wisconsin (R Nanchal); Northwestern Memorial Hospital (R Wunderink); Orlando Regional Medical Centre (E Jimenez); Washington Hospital Centre (A Ryan)

Oceania
Australia: Armadale Health Service (D Prince); Bendigo Hospital (J Edington); Canberra Hospital (F Van Haren); Flinders Medical Centre (A Bersten); Joondalup Health Campus (DJ Hawkins); Lismore Base Hospital (M Kilminster); Mater Adult Hospital (D Sturgess); Prince Charles Hospital, Brisbane (M Ziegenfuss); Royal Adelaide Hospital (S O' Connor); Royal Brisbane and Womens' Hospital (J Lipman); Royal Darwin Hospital (L Campbell); Royal Hobart Hospital (R Mcallister); Sir Charles Gairdner Hospital (B Roberts); The Queen Elizabeth Hospital (P Williams)
New Zealand: Auckland District Health Board (R Parke); Christchurch Hospital (P Seigne); Hawke's Bay Hospital (R Freebairn); Midcentral Health, Palmerston North Hospital (D Nistor); Middlemore Hospital (C Oxley); Wellington Hospital (P Young)

South and Central America
Argentina: Cemic (Centro De Educación Médica E Investigaciones Clínicas) (R Valentini); Fleni (N Wainsztein); Hospital Aleman (P Comignani); Hospital Central San Isidro (M Casaretto); Hospital Fernandez (G Sutton); Hospital Francisco Lopez Lima Area Programa General Roca (P Villegas); Sanatorio Allende (C Galletti); Sanatorio De La Trinidad Palermo (J Neira); Sanatorio Julio Corzo Rosario (D Rovira)
Belize: Karl Heusner Memorial Hospital and Belize Healthcare Partner (J Hidalgo)
Bolivia: Hospital Obrero No1 (F Sandi)
Brazil: Cias -Unimed Vitória (E Caser); Evangelical Hospital of Cachoeiro De Itapemirim (M Thompson); Hospital 9 De Julho (M D'agostino Dias); Hospital Alcides Carneiro (L Fontes); Hospital Das Clínicas Luzia De Pinho Melo (M Lunardi); Hospital Das Nações De Curitiba (N Youssef); Hospital De Base Famerp (S Lobo); Hospital De Clínicas De Niterói (R Silva); Hospital De Clínicas Padre Miguel (J Sales Jr); Hospital De Terapia Intensiva (L Madeira Campos Melo); Hospital Do Trabalhador (M Oliveira); Hospital Esperanca (M Fonte); Hospital Evangelico De Londrina (C Grion); Hospital Geral De Fortaleza (C Feijo); Hospital Geral De Roraima (V Rezende); Hospital Israelita Albert Einstein (M Assuncao); Hospital Mater Dei (A Neves); Hospital Meridional (P Gusman); Hospital Meridional (D Dalcomune); Hospital Moinhos De Vento (C Teixeira); Hospital Municipal Ruth Cardoso (K Kaefer); Hospital Nereu Ramos (I Maia); Hospital Pasteur (V Souza Dantas); Hospital Pro Cardiaco (R Costa Filho); Hospital Regional De Samambaia (F Amorim); Hospital Regional Hans Dieter Schmidt (M Assef); Hospital Santa Casa - Campo Mourão (P Schiavetto); Hospital Santa Paula (J Houly); Hospital São José Do Avaí (F Bianchi); Hospital São Lucas

Da Pucrs (F Dias); Hospital Sao Vicente De Paula (C Avila); Hospital São Vicente De Paulo (J Gomez); Hospital Saude Da Mulher (L Rego); Hospital Tacchini (P Castro); Hospital Unimed Costa Do Sol-Macae-Rj (J Passos); Hospital Universitário - Ufpb - João Pessoa (C Mendes); Hospital Universitário De Londrina (C Grion); Hospital Universitário São Francisco (G Colozza Mecatti); Santa Casa De Caridade De Diamantina (M Ferrreira); Santa Casa De Misericordia De Tatui (V Irineu); São Francisco De Paula Hospital (M Guerreiro)
Chile: Clinica Indisa (S Ugarte); Clinica Las Lilas (V Tomicic); Hospital Carlos Van Buren (C Godoy); Hospital Del Trabajador De Santiago (W Samaniego); Hospital El Pino (I Escamilla); Hospital Mutual De Seguridad (I Escamilla)
Colombia: Centro Medico Imbanaco (L Castro Castro); Clinica Colombia Cali (G Libreros Duque); Clínica Del Café (D Diaz-Guio); Clínica La Estancia S.A. (F Benítez); Clinica Medellin (A Guerra Urrego); Fundacion Clinica Shaio (R Buitrago); Hospital Santa Clara (G Ortiz); Hospital Universitario Fundación Santa Fe De Bogota (M Villalba Gaviria)
Costa Rica: Calderón Guardia Hospital (D Salas); Hospital Dr Rafael Angel Varlderon Guardia CCSS (J Ramirez-Arce)
Ecuador: Clinica La Merced (E Salgado); Hospital Eugenio Espejo (D Morocho); Hospital Luis Vernaza (J Vergara); Shdug Sistema Hospitalario Docente De La Universidad De Guayaquil (M Chung Sang)
El Salvador: General Hospital (C Orellana-Jimenez)
Guatemala: Hospital Centro Medico (L Garrido)
Honduras: Instituto Hondureño Del Seguro Social (O Diaz)
Martinique: Centre Hospitalier Universitaire De Fort-De-France (D Resiere)
Mexico: Centro Estatal De Cuidados Críticos (C Osorio); Centro Médico Nacional "20 De Noviembre" ISSSTE (A De La Vega); Fundacion Clinica Medica Sur (R Carrillo); Hospital San Jose TEC Monterrey (V Sanchez); Hospital 1o De Octubre, ISSSTE (A Villagomez); Hospital Español De Mexico (R Martinez Zubieta); Hospital General Ajusco Medio (M Sandia); Hospital General Guadalupe Victoria (M Zalatiel); Hospital Juarez De Mexico (M Poblano); Hospitalcivil De Guadalajara, Hspitaljuan I Menchaca (D Rodriguez Gonzalez); Instituto Mexicano Del Seguro Social (F Arrazola); Instituto Mexicano Del Seguro Social (L Juan Francisco); Instituto Nacional de Cancerología, México (SA Ñamendys-Silva); ISSSTE (R Guerra Moya); Medical Centre ISSEMYM Toluca (M Hernandez); Mixta (D Rodriguez Cadena); Secretaria De Salud Del Distrito Federal (I Lopez Islas)
Panama: Hospital Santo Tomás (C Ballesteros Zarzavilla); Social Security Hospital (A Matos)
Peru: Clinica Anglo Americana (I Oyanguren); ESSALUD (J Cerna); Hospital Nacional Dos De Mayo (R Quispe Sierra); Hospital Rebagliati (R Jimenez); Instituto Nacional De Enfermedades Neoplasicas (L Castillo)
Turks And Caicos Islands: Gulhane Medical Faculty (R Ocal); Izmir Atatürk Educational And Research Hosp. (A Sencan)
Uruguay: CAMS (S Mareque Gianoni); CASMU (A Deicas); Hospital Español Asse (J Hurtado); Hospital Maciel (G Burghi)
Venezuela: Centro Medico De Caracas (A Martinelli); Hospital Miguel Perez Carreño (I Von Der Osten)

South Asia
Afghanistan: MSF Trauma Hospital Kunduz (C Du Maine)
India: Amri Hospitals (M Bhattacharyya); Amri Hospitals Salt Lake (S Bandyopadhyay); Apollo Hospital (S Yanamala); Apollo Hospitals (P Gopal); Apollo Hospitals, Bhubaneswar (S Sahu); Apollo Speciality Hospital (M Ibrahim); Asian Heart Institute (D Rathod); Baby Memorial Hospital Ltd, Calicut, Kerala (N Mukundan); Batra Hospital & Mrc, New Delhi (A

Dewan); Bombay Hospital Institute of Medical Sciences (P Amin); Care Hospital (S Samavedam); Cims Hospital (B Shah); Columbia Asia Hospital, Mysore (G Gurupal); Dispur Hospitals (B Lahkar); Fortis Hospital (A Mandal); Fortis Hospital (Noida) (M Sircar); Fortis-Escorts Hospital, Faridabad (S Ghosh); Ganga Medical Centre & Hospital P Ltd. (V Balasubramani); Hinduja Hospital (F Kapadia); KDAH (S Vadi); Kerala Institute of Medical Sciences (KIMS, RMCC) (K Nair); Kalinga Institute of Medical Sciences (KIMS, DTEM) (S Tripathy); Kovai Medical Centre and Hospital (S Nandakumar); Medanta The Medicity, Gurgaon (J Sharma); Medica Superspecialty Hospitals (A Kar); Metro Heart Institute with Multispeciality (S Jha); Ruby Hall Pune (K Zirpe); Saifee Hospital (M Patel); Spandan Multispeciality Hospital (A Bhavsar); Tata Main Hospital (D Samaddar); Tata Memorial Hospital (A Kulkarni)
Pakistan: Aga Khan University (M Hashmi); Hearts International Hospital (W Ali); Liaquat National Hospital (S Nadeem)
Sri Lanka: Sri Jayewardenepura General Hospital (K Indraratna)

West Europe
Andorra: Hospital Nostra Senyora De Meritxell (A Margarit)
Austria: Akh Wien (P Urbanek); Allgemeines Und Orthopädisches Landeskrankenhaus Stolzalpe (J Schlieber); Barmherzige Schwestern Linz (J Reisinger); General Hospital Braunau (J Auer); Krankenhaus D. Barmherzigen Schwestern Ried I.I. (A Hartjes); Krankenhaus Floridsdorf (A Lerche); LK Gmünd-Waidhofen/Thaya-Zwettl, Standort Zwettl (T Janous); LKH Hörgas-Enzenbach (E Kink); LKH West (W Krahulec); University Hospital (K Smolle)
Belgium: AZ Groeninge Kortrijk (M Van Der Schueren); AZ Jan Palfijn Gent (P Thibo); AZ Turnhout (M Vanhoof); Bracops Anderlecht (I Ahmet); Centre Hospitalier Mouscron (P Gadisseux); CH Peltzer La Tourelle (P Dufaye); Chirec Edith Cavell (O Jacobs); CHR Citadelle (V Fraipont); CHU Charleroi (P Biston); CHU Mont-Godinne (A Dive); CHU Tivoli (Y Bouckaert); CHwapi (E Gilbert); Clinique Saint-Pierre Ottignies (B Gressens); Clinique-Maternité Sainte Elisabeth (E Pinck); Cliniques De L'Europe - St-Michel (V Collin); Erasme University Hospital (JL Vincent); Ghent University Hospital (J De Waele); Moliere Hospital (R Rimachi); Notre Dame (D Gusu); Onze Lieve Vrouw Ziekenhuis, Aalst (K De Decker); Ixelles Hospital (K Mandianga); Sint-Augustinus (L Heytens); St Luc University Hospital (UCL) (X Wittebole); UZ Brussel (H Spapen); Vivalia Site De Libramont (O Van Collie); VZW Gezondheidszorg Oostkust Knokke-Heist (W Vandenheede); ZNA Middelheim (P Rogiers)
Denmark: Herning Hospital (P Kolodzeike); Hjoerring Hospital (M Kruse); Vejle Hospital (T Andersen)
Finland: Helsinki University Central Hospital (V Harjola); Seinäjoki Central Hospital (K Saarinen)
France: Aix Marseille Univ, Hôpital Nord (M Leone); Calmette Hospital, Lille (A Durocher); Centre Hospitalier de Dunkerque (S Moulront); Centre Hospitalier Lyon Sud (A Lepape); Centre Hospitalo-Universitaire Nancy-Brabois (M Losser); CH Saint Philibert, Ghicl, Lille (P Cabaret); CHR De Dax (E Kalaitzis); CHU Amiens (E Zogheib); CHU Dijon (P Charve); CHU Dupuytren, Limoges (B François); CHU Nîmes (JY Lefrant); Centre Hospitalier De Troyes (B Beilouny); Groupe Hospitalier Est Francilien-Centre Hospitalier De Meaux (X Forceville); Groupe Hospitalier Paris Saint Joseph (B Misset); Hopital Antoine Béclère (F Jacobs); Hopital Edouard Herriot (B Floccard); Hôpital Lariboisère, APHP, Paris France (D Payen); Hopital Maison Blanche, Reims (A Wynckel); Hopitaux Universitaires de Strasbourg (V Castelain); Hospices Civils de Lyon (A Faure); CHU-Grenoble (P Lavagne); CHU-Nantes (T Lepoivre); Réanimation, CHU Lille (M Moussa); University Hospital Ambroise Paré (A

Vieillard-Baron); University Hospital Grenoble (M Durand); University Hospital of Marseille (M Gainnier); University of Nice (C Ichai)
Germany: Alexianer Krefeld Gmbh (S Arens); Charite Hochschulmedizin Berlin (C Hoffmann); Charite-University-Hospital, Berlin (M Kaffarnik); Diakoniekrankenhaus Henriettenstiftung Gmbh (C Scharnofske); Elisabeth-Krankenhaus Essen (I Voigt); Harlaching Hospital, Munich Municipal Hospital Group (C Peckelsen); Helios St. Johannes Klinik (M Weber); Hospital St. Georg Leipzig (J Gille); Klinik Hennigsdorf Der Oberhavel Kliniken Gmbh (A Lange); Klinik Tettnang (G Schoser); Klinikum "St. Georg" Leipzig (A Sablotzki); Klinikum Augsburg (U Jaschinski); Klinikum Augsburg (A Bluethgen); Klinikum Bremen-Mitte (F Vogel); Klinikum Bremen-Ost (A Tscheu); Klinikum Heidenheim (T Fuchs); Klinikum Links Der Weser Gmbh (M Wattenberg); Klinikum Luedenscheid (T Helmes); Krankenhaus Neuwerk (S Scieszka); Marienkrankenhaus Schwerte (M Heintz); Medical Centre Cologne Merheim (S Sakka); Schwarzwald-Baar Klinikum Villingen-Schwenningen (J Kohler); St. Elisabeth Krankenhaus Köln-Hohenlind (F Fiedler); St. Martinus Hospital Olpe (M Danz); Uniklinikum Jena (Y Sakr); Universitätsklinikum Tübingen (R Riessen); Universitätsmedizin Mainz (T Kerz); University Hospital Aachen, CPACC (A Kersten); University Hospital Aachen, DMIII (F Tacke); University Hospital Aachen, OIC (G Marx); University Hospital Muenster (T Volkert); University Medical Centre Freiburg (A Schmutz); University Medical Centre Hamburg-Eppendorf (A Nierhaus); University Medical Centre Hamburg-Eppendorf (S Kluge); University Medicine Greifswald (P Abel); University of Duisburg-Essen (R Janosi); University of Freiburg (S Utzolino); University clinic Ulm (H Bracht); Vivantes Klinikum Neukoelln (S Toussaint)
Greece: Ahepa University Hospital (M Giannakou Peftoulidou); Athens University (P Myrianthefs); Athens University Medical School (A Armaganidis); Evangelismos Hospital (C Routsi); General Hospital of Chania, Crete (A Xini); Hippokration General Hospital, Thessaloniki (E Mouloudi); General hospital of Velos (I Kokoris); Lamia General Hospital (G Kyriazopoulos); Naval and Veterans Hospital (S Vlachos); Papanikolaou General Hospital (A Lavrentieva); University Hospital Alexandroupolis (P Partala); University of Ioannina (G Nakos)
Iceland: Landspitali University Hospital (A Moller); Landspitali University Hospital Fossvogur (S Stefansson)
Ireland: Cork University Hospital (J Barry); Mercy University Hospital (R O'Leary); Mid Western Regional Hospital Complex (C Motherway); Midland Regional Hospital Mullingar, Co Westmeath (M Faheem); St. Vincent's University Hospital (E Dunne); Tallaght Hospital (M Donnelly); University Hospital Galway (T Konrad)
Italy: Anesthesiology and Intensive Care (E Bonora); AO Ospedale Niguarda Ca' Granda (C Achilli); Azienda Ospedaliera Di Padova (S Rossi); Azienda Ospedaliero Universitaria Policlinico Vittorio Emanuele (G Castiglione); Careggi Teaching Hospital (A Peris); Clinicized Hospital SS Annunziata - Chieti (D Albanese); Fondazione IRCCS Ca' Granda Ospedale Maggiore Policlinico, Milano; University of Milan (N Stocchetti); H San Gerardo - Monza (G Citerio); ICU "Ceccarini" Hospital Riccione (L Mozzoni); IRCCS Centro Cardiologico Monzino (E Sisillo); IRCCS Centro Di Riferimento Oncologico Della Basilicata (P De Negri); IRCCS Fondazione Ca' Granda - Ospedale Maggiore Policlinico (M Savioli); Ospedale Belcolle Viterbo (P Vecchiarelli); Ospedale Civile Maggiore - A.O.U.I Verona (F Puflea); Ospedale Civile Maggiore - A.O.U.I Verona (V Stankovic); Ospedale Di Circolo E Fondazione Macchi - Varese (G Minoja); Ospedale Di Trento - Azienda Provinciale Per I Servizi Sanitari Della Provincia Autonoma Di Trento (S Montibeller); Ospedale Orlandi (P Calligaro); Ospedale Regionale U.Parini-Aosta (R Sorrentino); Ospedale San Donato Arezzo (M Feri); Ospedale San Raffaele (M Zambon); Policlinico G.B. Rossi - A.O.U.I Verona (E Colombaroli); Policlinico University of Palermo (A Giarratano); Santa Maria Degli Angeli

Hospital (T Pellis); Saronno Hospital (C Capra); Università Cattolica Del Sacro Cuore (M Antonelli); University Catania, Italy (A Gullo); University of Florence, Florence (C Chelazzi); University of Foggia (A De Capraris); University of Milano-Bicocca, San Gerardo Hospital (N Patroniti); University of Modena (M Girardis); University of Siena (F Franchi); University of Trieste (G Berlot)
Malta: Mater Dei Hospital (M Buttigieg)
Netherlands: Albert Schweitzer Hospital (H Ponssen); Antoni Van Leeuwenhoek Ziekenhuis (J Ten Cate); Atrium Medisch Centrum Parkstad (L Bormans); Bovenij Hospital (S Husada); Catharina Hospital Eindhoven (M Buise); Erasmus University Medical Centre (B Van Der Hoven); Martiniziekenhuis Groningen (A Reidinga); Medical Centre Leeuwarden (M Kuiper); Radboud University Nijmegen Medical Centre (P Pickkers); Slotervaart Ziekenhuis Amsterdam (G Kluge); Spaarne Ziekenhuis (S Den Boer); University Medical Centre Utrecht (J Kesecioglu); Ziekenhuis Rijnstate (H Van Leeuwen)
Norway: Haukeland University Hospital (H Flaatten); St Olavs Hospital, Trondheim University Hospital (S Mo)
Portugal: Centro Hospitalar Cova Da Beira (V Branco); Centro Hospitalar Do Porto (F Rua); Centro Hospitalar Do Tâmega E Sousa (E Lafuente); Centro Hospitalar Gaia/Espinho, Epe (M Sousa); Centro Hospitalar Médio Tejo (N Catorze); Centro Hospitalar Tondela-Viseu (M Barros); Faro Hospital (L Pereira); Hospital Curry Cabral (A Vintém De Oliveira); Hospital Da Luz (J Gomes); Hospital De Egas Moniz - Chlo (I Gaspar); Hospital De Santo António, Centro Hospitalar Do Porto (M Pereira); Hospital Divino Espírito Santo, Epe (M Cymbron); Hospital Espirito Santo - Évora Epe (A Dias); Hospital Garcia Orta (E Almeida); Hospital Geral Centro Hospitalar E Universitario Coimbra (S Beirao); Hospital Prof. Doutor Fernando Fonseca Epe (I Serra); Hospital São Bernardo (R Ribeiro); Hospital Sao Francisco Xavier, Chlo (P Povoa); Instituto Portugues De Oncologia Francisco Gentil, Porto (F Faria); Santa Maria Hospital (Z Costa-E-Silva); Serviço De Saúde Da Região Autonóma Da Madeira (J Nóbrega); UCIP (F Fernandes); ULS - Castelo Branco (J Gabriel)
Slovenia: General Hospital Celje (G Voga); General Hospital Izola (E Rupnik); General Hospital Novo Mesto (L Kosec); Oncological Institute (M Kerin Povšic); Ukc Maribor (I Osojnik); University Clinic of Respiratory and Allergic Diseases (V Tomic); University Clinical Centre Maribor (A Sinkovic)
Spain: CH Salamanca (J González); Clinic Hospital (E Zavala); Complejo Hospitalario De Jaén (J Pérez Valenzuela); Complejo Hospitalario De Toledo (L Marina); Complexo Hospitalario Universitario De Ourense (P Vidal-Cortés); Complexo Hospitalario Universitario De Vigo (P Posada); Corporación Sanitaria Parc Tauli (I Martin-Loeches); Cruz Roja Hospital (N Muñoz Guillén); H Vall Hebron (M Palomar); HGGC Dr Negrín (J Sole-Violan); Hospital Clinic (A Torres); Hospital Clinico San Carlos (M Gonzalez Gallego); Hospital Clínico Universitario De Valencia (G Aguilar); Hospital Clínico Universitario Lozano Blesa (R Montoiro Allué); Hospital Clinico Valencia (M Argüeso); Hospital De La Ribera (M Parejo); Hospital De Sagunto (M Palomo Navarro); Hospital De San Juan De Alicante (A Jose); Hospital De Torrejon De Ardoz (N Nin); Hospital Del Mar (F Alvarez Lerma); Hospital Del Tajo (O Martinez); Hospital General Universitario De Elche (E Tenza Lozano); Hospital General Universitario Gregorio Marañon (S Arenal López); Hospital General Universitario Gregorio Marañon (M Perez Granda); Hospital General Universitario Santa Lucía (S Moreno); Hospital Germans Trias I Pujol (C Llubia); Hospital Infanta Margarita (C De La Fuente Martos); Hospital Infanta Sofia (P Gonzalez-Arenas); Hospital J.M. Morales Meseguer (N Llamas Fernández); Hospital J.M. Morales Meseguer (B Gil Rueda); Hospital Marina Salu. Denia. Alicante. (I Estruch Pons); Hospital Nuestra Señora Del Prado, Talavera De La Reina, Toledo. España (N Cruza); Hospital San Juan De Dios Aljarafe (F Maroto); Hospital Sas of Jerez (A Estella); Hospital Son Llatzer (A Ferrer); Hospital Universitario

Central De Asturias (L Iglesias Fraile); Hospital Universitario Central De Asturias (B Quindos); Hospital Universitario De Alava, Santiago (A Quintano); Hospital Universitario De Basurto, Bilbao (M Tebar); Hospital Universitario de Getafe (P Cardinal); Hospital Universitario De La Princesa (A Reyes); Hospital Universitario de Tarragona Joan Xxiii (A Rodríguez); Hospital Universitario Del Henares (A Abella); Hospital Universitario Fundación Alcorcón (S García Del Valle); Hospital Universitario La Paz (S Yus); Hospital Universitario La Paz (E Maseda); Hospital Universitario Rio Hortega (J Berezo); Hospital Universitario San Cecilio (Granada) (A Tejero Pedregosa); Hospital Virgen Del Camino (C Laplaza); Mutua Terrassa University Hospital (R Ferrer); Rão Hortega University Hospital (J Rico-Feijoo); Servicio Andaluz De Salud (M Rodríguez); University Okalingaf Navarra (P Monedero)
Sweden: Karolinska University Hospital And Karolinska Institute (K Eriksson); Sunderby Hospital, Luleå (D Lind)
Switzerland: Hôpital Intercantonal De La Broye (D Chabanel); Hôpital Neuchâtelois - La Chaux-De-Fonds (H Zender); Lindenhofspital (K Heer); Regionalspital Surselva Ilanz (Gr) Schweiz (B Frankenberger); University Hospital Bern (S Jakob); Zentrum Für Intensivmedizin (A Haller)
United Kingdom: Alexandra Hospital Redditch (S Matthew); Blackpool Teaching Hospitals (R Downes); Brighton And Sussex University Hospitals (C Barrera Groba); Cambridge University Hospitals NHS Foundation Trust (A Johnston); Charing Cross Hospital (R Meacher); Chelsea & Westminster Hospital (R Keays); Christie Foundation Trust (P Haji-Michael); County Hospital, Lincoln (C Tyler); Craigavon Area Hospital (A Ferguson); Cumberland Infirmary (S Jones); Darent Valley Hospital (D Tyl); Dorset County Hospital (A Ball); Ealing Hospital NHS Trust (J Vogel); Glasgow Royal Infirmary (M Booth); Gloucester Royal Hospital (P Downie); The Great Western Hospital, Swindon (M Watters); Imperial College Healthcare NHS Trust (S Brett); Ipswich Hospital NHS Trust (M Garfield); James Paget University Hospital NHS Foundation Trust (L Everett); King's College Hospital (S Heenen); King's Mill Hospital (S Dhir); Leeds Teaching Hospitals NHS Trust (Z Beardow); Lewisham Healthcare NHS Trust (M Mostert); Luton and Dunstable Hospital NHS Trust (S Brosnan); Medway Maritime Hospital (N Pinto); Musgrove Park Hospital (S Harris); Nevill Hall Hospital (A Summors); Pilgrim Hospital (A Norton); Pinderfields Hospital, Mid Yorkshire NHS Trust (A Rose); Plymouth Hospitals NHS Trust (R Appelboam); Princess Royal Hospital Telford (O Davies); Royal Bournemouth Hospital (E Vickers); Royal Free Hampstead NHS Foundation Trust (B Agarwal); Royal Glamorgan Hospital (T Szakmany); Royal Hampshire County Hospital (S Wimbush); Royal Liverpool University Hospital (I Welters); Royal London Hospital, Barts Health NHS Trust (R Pearse); Royal Shrewsbury Hospital (R Hollands); Royal Surrey County Hospital (J Kirk-Bayley); St Georges Healthcare (N Fletcher); Surrey & Sussex Healthcare Trust (B Bray); University College Hospital (D Brealey); University Hospital of South Manchester (P Alexander); Western Infirmary (S Henderson); Whittington Hospital (C Hargreaves); Wirral University Teaching Hospital (H Black); Yeovil District Hospital (K Gowda)
